# Dual short wavelength infrared transillumination/reflectance mode imaging for caries detection

**DOI:** 10.1117/1.JBO.26.4.043004

**Published:** 2021-01-29

**Authors:** Yihua Zhu, Marwa Abdelaziz, Jacob Simon, Oanh Le, Daniel Fried

**Affiliations:** University of California, San Francisco, San Francisco, California, United States

**Keywords:** short wavelength infrared imaging, caries detection, reflectance, transillumination

## Abstract

**Significance:** We have developed a clinical probe capable of acquiring near-simultaneous short-wavelength infrared (SWIR) reflectance and occlusal transillumination images of lesions on tooth proximal and occlusal surfaces. We hypothesize that dual images will aid in differentiating between shallow and deep occlusal lesions and reduce the potential of false positives (FPs).

**Aim:** The aim of this study was to test the performance of the dual reflectance and occlusal transillumination probe on extracted teeth prior to commencing clinical studies.

**Approach:** The dual probe was 3D printed and the imaging system uses an InGaAs camera and broadband superluminescent diode light sources that emit broadband light at 1300 nm for occlusal transillumination and 1600-nm light for cross-polarization reflectance. The diagnostic performance of the dual probe was assessed using 120 extracted teeth with approximal and occlusal lesions. Reflectance and transillumination images were fused into single images to enhance the contrast between sound and lesion areas. The lesion contrast in both modes did not increase significantly with either the lesion depth or the distance from the occlusal surface for approximal lesions. In addition, the diagnostic performance of radiography, the individual reflectance and transillumination images, dual images, and fused images were compared using micro-computed tomography as the gold standard.

**Results:** Reflectance imaging at 1600 nm yielded the highest diagnostic accuracy for lesions on both occlusal and proximal surfaces while radiography yielded the lowest number of FPs.

**Conclusion:** This study demonstrates that simultaneous acquisition of both reflectance and transillumination SWIR images is possible with a single clinical device.

## 1 Introduction

Short-wavelength infrared (SWIR) and near-IR imaging (NIR) methods have been under development for almost 20 years for use in dentistry and several NIR clinical devices are now available commercially. Due to the high transparency of enamel at longer wavelengths, novel imaging configurations are feasible in which the tooth can be imaged from the occlusal surface after shining light at and below the gum line, which we call occlusal transillumination.^1^^,^^2^ Approximal lesions, the lesions located at the proximal contact points in between teeth, can be imaged via occlusal transillumination of the proximal contact points between teeth and by directing SWIR light below the crown while imaging the occlusal surface.^2^^–^^4^ The latter approach is capable of imaging occlusal lesions as well with high contrast.^1^^,^^2^^,^^5^^–^^8^ In 2010, it was demonstrated that approximal lesions that appeared on radiographs could be detected *in vivo* with SWIR imaging at 1310 nm with similar sensitivity^2^ and that occlusal transillumination could be employed clinically. This was the first step in demonstrating the clinical potential of NIR and SWIR imaging for approximal caries detection. In the most recent clinical study in 2016,^9^ SWIR transillumination and SWIR reflectance imaging probes were used to screen premolar teeth scheduled for extraction for caries lesions at wavelengths >1300  nm. The teeth were collected and sectioned into 200-μm slices and examined with polarized light microscopy (PLM) and transverse microradiography that served as the gold standard. In addition, extra-oral radiographs were taken of teeth and the diagnostic performance of SWIR imaging was compared with radiography. The sensitivity of the combined SWIR imaging probes was significantly higher (P<0.05) than radiographs for both occlusal and approximal lesions *in vivo*. It was anticipated that SWIR methods would be more sensitive than radiographs for occlusal lesions since the radiographic sensitivity for occlusal lesions is extremely poor; however, the sensitivity was also much higher for approximal lesions than radiography, 0.53 versus 0.23. In addition, the sensitivity of each individual SWIR method was either individually equal to or higher than radiography.

Several shorter wavelength NIR devices that utilize either reflectance and transillumination imaging have been introduced commercially operating at 830 and 780 nm.^10^^–^^14^ The shorter wavelength allows the use of less expensive, silicon-based detectors. However, longer wavelength SWIR light has significant advantages. The use of shorter wavelength 830-nm NIR light was first investigated almost 20 years ago.^3^ The contrast was significantly lower than at 1300 nm and simulated lesions could not be imaged through the full thickness of enamel.^3^ It is also important to point out that stains interfere significantly at 780 nm^15^ and the contrast between sound and demineralized enamel is markedly higher at wavelengths beyond 1400 nm in reflectance measurements.^15^^–^^17^

Epidemiological data gathered from the National Health and Nutritional Survey^18^^,^^19^ and Dental Practice-Based Research Network^20^^–^^22^ indicates that nearly one third of all patients have a “questionable” occlusal carious lesion (QOC) located on a posterior tooth. QOCs are given the name questionable because clinicians’ lack instrumentation capable of measuring the depth of pit and fissure lesions and determining if the dental decay has reached the underlying dentin. Bitewing radiographs are not sensitive enough to detect most occlusal lesions, and visible diagnosis is confounded by stain trapped in the occlusal anatomy.^15^ Prior *in vitro* studies attempted to combine SWIR reflectance and transillumination measurements to estimate QOC depth and severity.^7^^,^^23^^,^^24^ Salsone et al.^25^ and Zakian et al.^26^ used multiple wavelengths of SWIR hyperspectral reflectance images to estimate the severity of occlusal lesions. Since, multispectral SWIR reflectance, and transillumination experiments have demonstrated that the tooth appears darker at wavelengths coincident with increased water absorption, multispectral images can be used to produce increased contrast between different tooth structures such as sound enamel and dentin, dental decay, and composite restorative materials.^26^^–^^28^ Combining measurements from different SWIR imaging wavelengths and comparing them with concurrent measurements acquired by complementary imaging modalities should provide improved assessment of lesion depth and severity.

Both the reflectance and the occlusal transillumination probes sample light that is emitted from tooth occlusal surfaces, therefore it is feasible to combine both methods into a single probe that can be positioned above the tooth for rapid clinical screening. Different illumination wavelengths can be used that are optimized for each imaging mode, namely, SWIR wavelengths >1400  nm for reflectance and 1300 nm for transillumination. Simon et al.^29^ built a benchtop simultaneous SWIR reflectance and transillumination system with tunable filters that ranged from 830 to 1700 nm and showed that the combined images have potential for the diagnosis of QOCs. In a study, the following year using the same benchtop system and simulated approximal lesions showed that multispectral combined images can potentially be used to improve the differentiation of cavitated and noncavitated approximal lesions.^30^ In a third study carried out in 2018, Simon et al.^24^ used the same benchtop system to image 37 extracted teeth with occlusal caries lesions and sectioned the teeth and measured the lesion depth with PLM. This 2018 study showed that the contrast of reflectance at 1500 to 1700 nm correlated with the lesion depth while the transillumination at 1200 to 1700 nm showed no correlation. The lesion width, the lesions’ buccal-lingual dimension along the fissure, showed a positive correlation with lesion depth for reflectance and transillumination. These initial investigations demonstrated the potential of acquiring simultaneous multiwavelength SWIR reflection and transillumination images to improve the detection of caries lesions on both occlusal and proximal surfaces. We hypothesize that the greatest utility of a combined SWIR reflectance and transillumination clinical probe will be to reduce false positives (FPs) since it is unlikely that confounding structural features or specular reflection are going to be present in both reflectance and transillumination images. In addition, the dual probe will provide complementary diagnostic information about lesion severity to help discriminate early superficial lesions on tooth surfaces from deeply penetrating lesions.

In this study, a system for the acquisition of simultaneous SWIR reflectance and transillumination system suitable for clinical use was developed and tested on 120 extracted teeth with occlusal and approximal lesions using micro-computed tomography (μCT) as a gold standard.

## 2 Materials and Methods

### 2.1 Sample Preparation

Teeth with no identifiers were collected from patients in the San Francisco Bay area and Geneva Switzerland with approval from the UCSF Committee on Human Research. Extracted teeth (n=120) were selected with occlusal and approximal lesions for this study. Teeth were sterilized using gamma radiation and stored in 0.1% thymol solution to maintain tissue hydration and prevent bacterial growth. Then, samples were mounted in black orthodontic acrylic blocks from Great Lakes Orthodontics (Tonawanda, New York) and imaged with digital radiographs using a CareStream 2200 System from Kodak (Rochester, New York) operating at 60 kV.

All teeth were imaged using μCT with a 10-μm resolution. A Scanco μCT 50 from Scanco USA (Wayne, Pennsylvania) located at the UCSF Bone Imaging Core Facility was used to acquire the images. Visible color images of the samples were acquired using a USB microscope, Model AM7915MZT from AnMO Electronics Corp. (New Taipei City, Taiwan) with extended depth of field and cross polarization. The digital microscope captures 5 mega-pixel (2952×1944) color images.

### 2.2 Design and Fabrication of the Dual SWIR probe

**Fig. 1:**
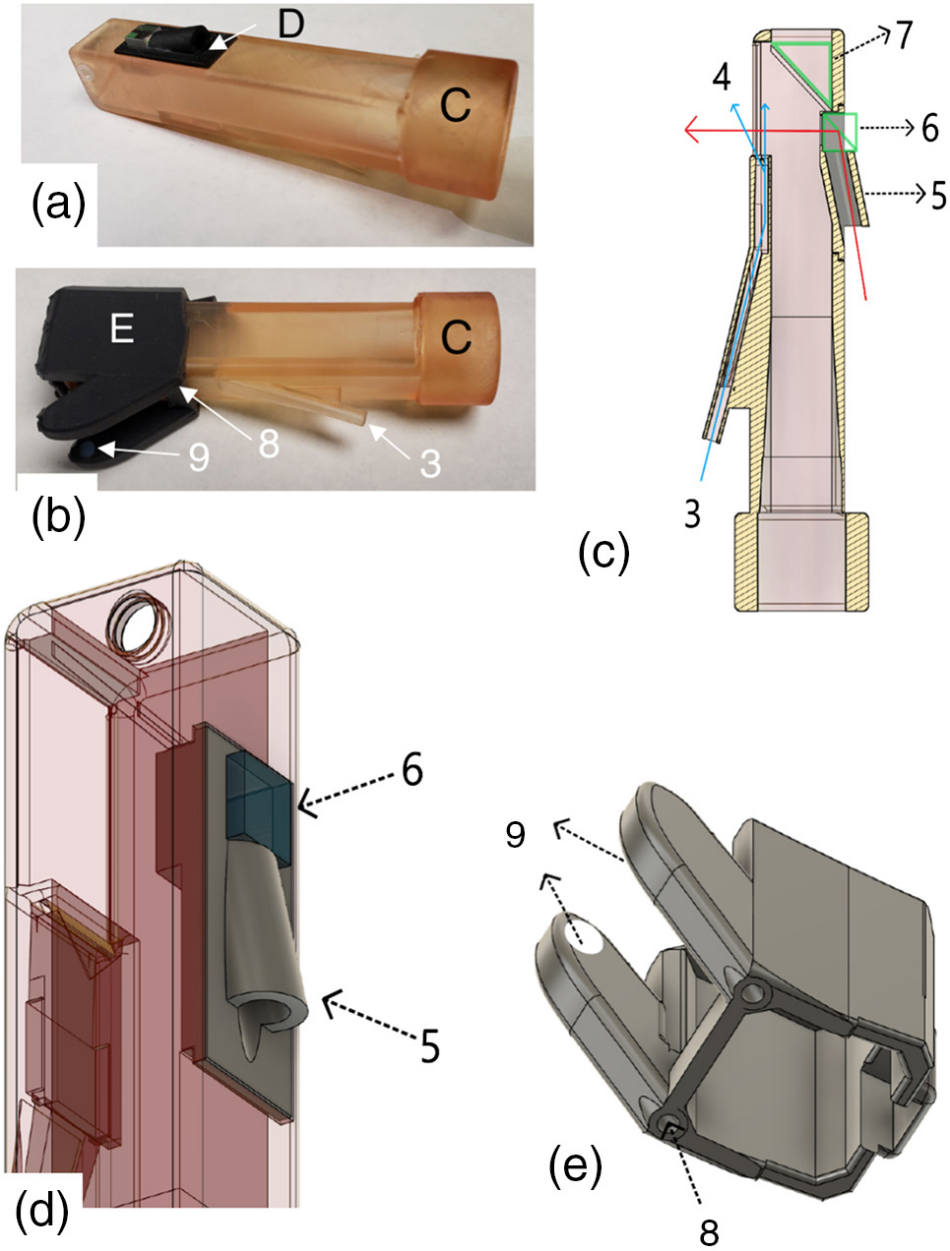
(a) 3D printed handpiece for reflectance with insert (d) made of black resin with a polarizing beamsplitter cube (6); (b) Handpiece with attachment for transillumination (e). (c) Handpiece cross-sectional view; the reflectance fiber is inserted through the port at (5) from the back of the polarization chamber, and light is polarized (6) and delivered to the tooth occlusal surface as shown by the red arrow. Air enters the nozzle at (3) and is directed toward the tooth surface at (4) as shown by the blue arrows. Reflected and transmitted light from the tooth is directed to the camera by a right angle polished surface (7). The insert for the polarizing beamsplitter cube (d) is snapped to the back of the handpiece. Transillumination fibers are inserted into the two ports (8) at the rear of the transillumination appliance (e) and into the Teflon plugs (9) at the end of each probe arm. The transillumination appliance (e) is placed over the end of the reflectance attachment as shown in (b) and Fig. 2.

The dual probe was designed in Fusion 360 from Autodesk (San Francisco, California). The dual probe design consists of a handpiece with reflectance and a transillumination attachment, shown in Figs. 1(a)–1(c). The handpiece incorporated an insert [Fig. 1(d)] to attach the optical fiber for reflectance along with a 5-mm Thorlabs (Newton, New Jersey) polarizing beam splitter cube. The insert utilized black resin to reduce artifacts from unwanted scattering in the handpiece. A cross-sectional view of the reflectance probe body is shown in Fig. 1(c). Light from a fiber optic cable travels from the back of the handpiece to the insert [Fig. 1(d)] and is directed at a right angle by the polarizing beam splitter cube (6) toward the tooth surface as shown by the path of the red arrow in Fig. 1(c). Reflected and transmitted light from the tooth is reflected off a polished aluminum surface (7) attached at a 45-deg angle at the distal end of the handpiece. There is an air nozzle positioned on the bottom of the probe (3) directed toward the aluminum reflector to prevent fogging of the aluminum surface. The air nozzle can also be used to dry the tooth surface to increase lesion contrast and potentially assess lesion activity.^31^^–^^33^

The transillumination attachment [Fig. 1(e)] attaches to the distal end of the handpiece as shown in Fig. 1(b). It was fabricated with Formlabs (Boston, Massachusetts) Flexible Resin. The flexible resin can tolerate moderate stretch and compression, making it an ideal choice for imaging teeth with different shapes and sizes. The transillumination optical fibers enter each arm of the attachment and are inserted into the two ports at the rear of the appliance (8) as shown in Figs. 1(b) and 1(e) and into Teflon plugs (9). The Teflon plugs diffusely spread the light from the optical fiber into the tooth on the buccal and lingual surfaces below the dentinal enamel junction and into the gingival tissues when used clinically. They were fabricated from 1/8″ diameter Teflon rod cut to a length of 3 mm. The separation of each arm is 1 cm and they are flexible and can stretch to almost 2 cm to accommodate larger teeth.

The handpiece was fabricated using a Formlabs Form 3 Low Force Stereolithography 3D printer. The final design is exported as a Standard Tessellation Language (STL) file and transferred to Formlabs PreForm to generate supports for a final 3D printing scheme. A spatial resolution of 100  μm was used for all prints. Formlabs Dental SG Resin was used for printing the reflectance probe to provide biocompatibility and autoclavability. After 3D printing, handpiece was transferred to Formlabs Form Wash to rinse off the resin residue with isopropyl alcohol for 5 min and cure for 30 min at 60°C in the Form Cure. The attachment [Fig. 1(d)] housing the 5-mm polarization beam splitting (PBS) cube was printed using Formlabs black resin. After curing, supports are removed with a clip and surfaces are trimmed with water sanding technique to remove sharp edges and achieve smooth finishing.

To assemble the dual SWIR probe, the insert with the PBS cube [Fig. 1(d)] is attached to the top of the handpiece. The aluminum reflector (7) is attached to the distal end of the handpiece and held in place with a set screw. The transillumination attachment [Fig. 1(e)] slides on top of the handpiece and securely locks the insert with the beamsplitter cube (6) in place.

### 2.3 Image Acquisition and Analysis

**Fig. 2:**
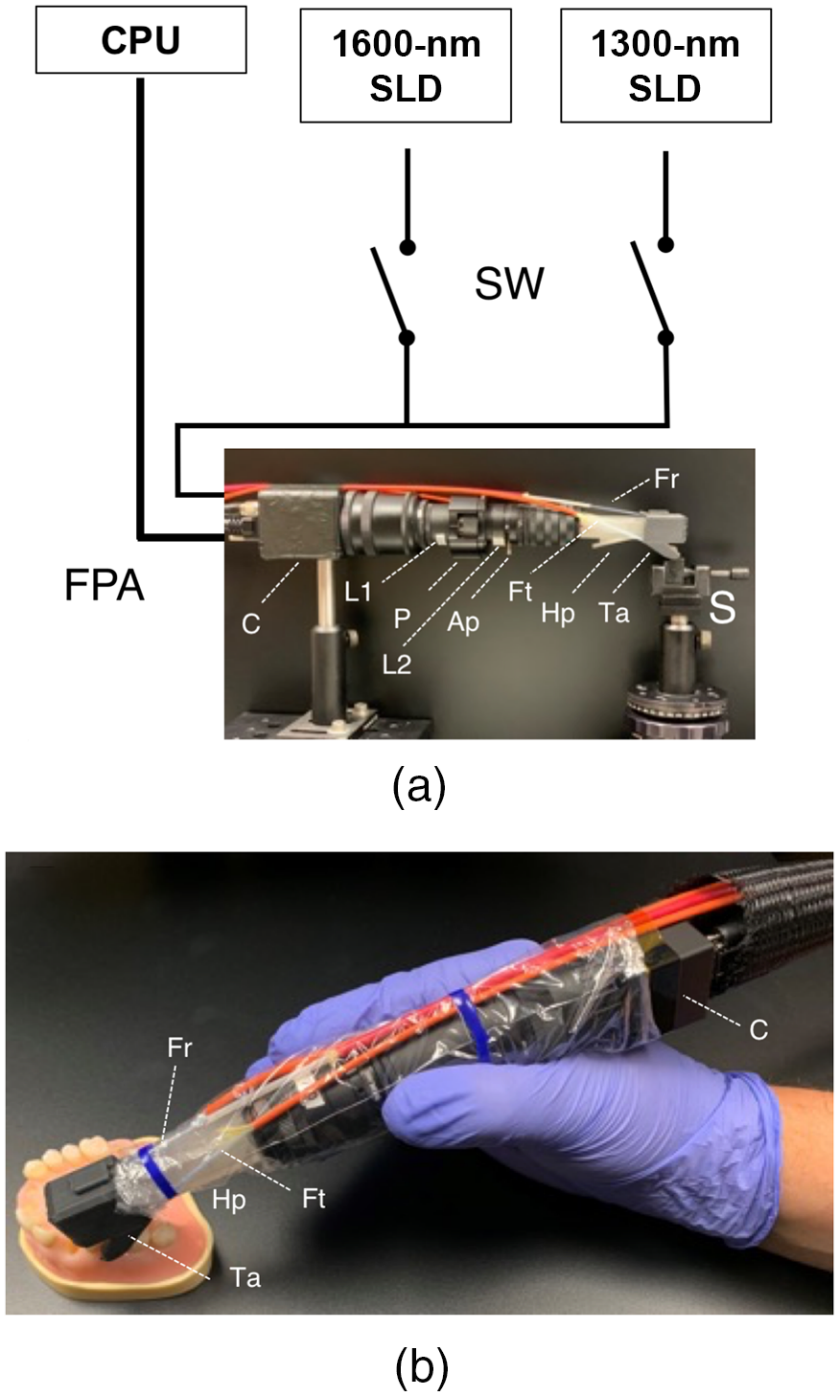
(a) Diagram of dual imaging system which employs a computer, two SLDs, two fiber optic switches (SW), and an InGaAs FPA with external optics and handpiece which is position over a sample tooth (S). The camera (C), 100-mm lens (L1), polarizer (P), 60-mm lens (L2), adjustable aperture (Ap), reflectance handpiece (Hp), and transillumination attachment (Ta) are shown along with the orange optical fibers for reflectance (Fr) and transillumination (Ft). (b) The handheld dual imaging handpiece equipped with the smaller InGaAs camera that will be used for clinical imaging.

The SWIR reflectance images were captured using a Model GA1280J (Sensors Unlimited, Princeton, New Jersey) camera with a 1280×1024  pixel format, a 15-μm pixel pitch, and a bit depth of 12-bit. Two 1-in. diameter planoconvex antireflection coated lenses of 60- and 100-mm focal length along with an adjustable aperture were placed between the handpiece and the InGaAs camera to provide a field of view of $11\times 11\;{\mathrm{mm}}^{2}$ at the focus plane. A low-OH optical fiber of 1-mm diameter was used to deliver light from a 1604-nm superluminescent diode (SLD), Model ESL 1620-2111 from Exalos (Schlieren, Switzerland) with an output of 17 mW and a bandwidth of 46 nm. The intensity delivered to the tooth was 5 mW. The transillumination light is delivered through two 0.4-mm diameter low-OH optical fibers. A 1314-nm SLD, Model DL-CS3452A-FP 1620-2111 from Denselight (Singapore) with an output of 48 mW and a bandwidth of 33 nm was used as the source for transillumination. A 50/50 beamsplitter was used to deliver light to each arm for transillumination. The output intensity of each arm was set at 10 mw before entering the teflon plugs. The reflectance and transillumination light sources output were controlled via two OSW12(22) MEMS fiber optic switches from Thorlabs. The camera and the optic switch were controlled in custom authored programs in LabView (Austin, Texas). The two optical switches alternate between the 1300-nm light source for transillumination and the 1600-nm source for reflectance allowing near-simultaneous acquisition at a rate of 30 Hz. A diagram of the setup along with an image of the with imaging optics and the assembled dual probe is shown in Fig. 2(a). Figure 2(a) shows the larger Model GA1280J InGaAs camera that was used to acquire the *in vitro* images used in this study. The probe can also be used with the smaller 640×480  pixel SWIR camera (SU640CSX) measuring only $32\times 32\times 28\;{\mathrm{mm}}^{3}$ from Sensors Unlimited (Princeton, New Jersey) that is better suited for clinical imaging and it is shown in Fig. 2(b) with the assembled probe.

The samples were dried of excess water with an air nozzle before imaging due to the strong water absorption at 1600 nm.^4^ Image processing of the images was performed by custom scripts written using MATLAB from Mathworks (Natick, Massachusetts). The acquired 12-bit images (4096) were converted to 16-bit (65536) by multiplying by 16 and subtracting 1 to facilitate processing using MATLAB. Lesion regions of interest (ROIs) were identified from the transillumination/reflectance images. Co-registration was unnecessary since they were acquired near simultaneously by the same imaging optics. Sound areas for comparison were chosen from areas surrounding the lesion. This better represents the contrast between the lesion and sound tissues that would be viewed by the clinician. The contrast was calculated for each lesion using the formula $(I_{L}-I_{S})/I_{L}$ for reflectance images and $(I_{S}-I_{L})/I_{S}$ for transillumination images, where $I_{L}$ is the average intensity in the lesion area ROI, and $I_{S}$ is the average intensity in the sound ROI.^5^ Lesion areas were confirmed using the μCT images.

In addition to calculating the lesion contrast in transillumination and reflectance images, a dual fusion mode combining the two images was created in MATLAB and included in the analysis utilizing the weight, or fraction of the intensity applied from each image. The dual fusion mode is a weighted linear combination of the intensities of the inverted transillumination ($I_{\mathrm{Tinv}}=65535-I_{T}$) and the reflectance ($I_{R}$) 16-bit images. The lesion contrast in fusion mode was calculated using $\alpha I_{\mathrm{Tinv}}+bI_{R}$, with alpha (α) ranging from 0 to 1, where α=1−b.

### 2.4 Scoring of Digital Radiographs and SWIR Images by Clinicians

Three clinical examiners participated in the study, two examiners had several years of research experience with SWIR imaging and one examiner had no prior experience with SWIR imaging. A training set was used to standardize the knowledge of the three examiners prior to the study. All examiners were presented with radiographs and SWIR images of 120 teeth and asked to score the occlusal, mesial approximal, and distal approximal surfaces of each tooth. At least one of the examiners indicated that at least one of the images for 14 of the teeth was unreadable. Therefore 106 sets of images with four different imaging methods (transillumination, reflectance, dual, and both) were evaluated. The order of the images was randomized for each of the four testing sets among the three examiners. The examiners were asked to provide a binary score (lesion or no lesion) for each of the three surfaces. Diagnostic accuracy was calculated using (TP + TN)/(TP + TN + FP + FN) where TP, TN, FP, and FN are true positives, true negatives, false positives and false negative, respectively. μCT was used as the gold standard. The percent agreement for each tooth was calculated, and then the average % agreement was calculated for all the samples to represent the study interrater reliability. Clinician scores of interrater reliability (percent agreement), accuracy, and FP were calculated for the four SWIR image types and the radiographs, and the values are tabulated in Table 2.

## 3 Results

**Fig. 3:**
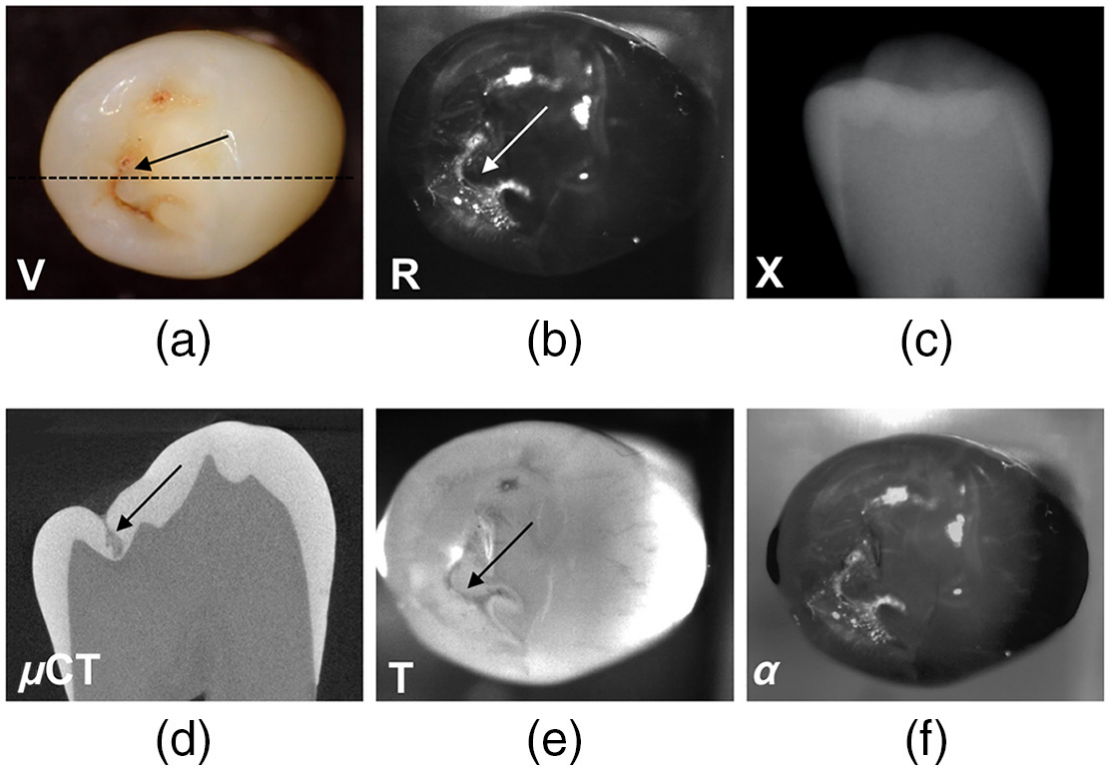
(a) Visible; (b) SWIR reflectance at 1600 nm; (c) dental radiographic; (d) μ-CT, (e) SWIR transillumination at 1300 nm; and (f) combined R + T fusion (α=0.4) images of a tooth with an occlusal lesion. Arrows point to the occlusal lesion and the dotted line in (a) shows the orientation of the slice in the μCT image.

Several images from a tooth with an occlusal lesion in a fissure that penetrates almost to the underlying dentin are shown in Fig. 3. The visible image of the occlusal surface [Fig. 3(a)] shows a mandibular premolar with red/brown staining in the central fissure and mesial pit, however, there is no evidence of increased white (value) areas that are indicative of visible demineralization on the tooth surface. The SWIR reflectance image [Fig. 3(b)] shows with high contrast increased light scattering from the stained fissure and pit indicating that the enamel is demineralized in these areas. There are also some other areas of high intensity, particularly on the buccal cusp tip that may be due to demineralization but is more likely specular reflection from the surface. There is no decay visible in the radiograph [Fig. 3(c)] while the μCT slice clearly shows that the V-shaped lesion in the fissure has penetrated almost through the enamel. The decay in the fissure and the pit is also detectable in the transillumination image [Fig. 3(e)] increasing the probability that those areas are actually demineralized and not due to an optical anomaly. The bright spot originating from the buccal cusp tip in the reflectance image is not visible in the transillumination image and is likely an area of specular reflection. The fusion image [Fig. 3(f)] shows the lesion areas with high contrast, however, it also highlights areas of suspected specular reflection.

**Fig. 4:**
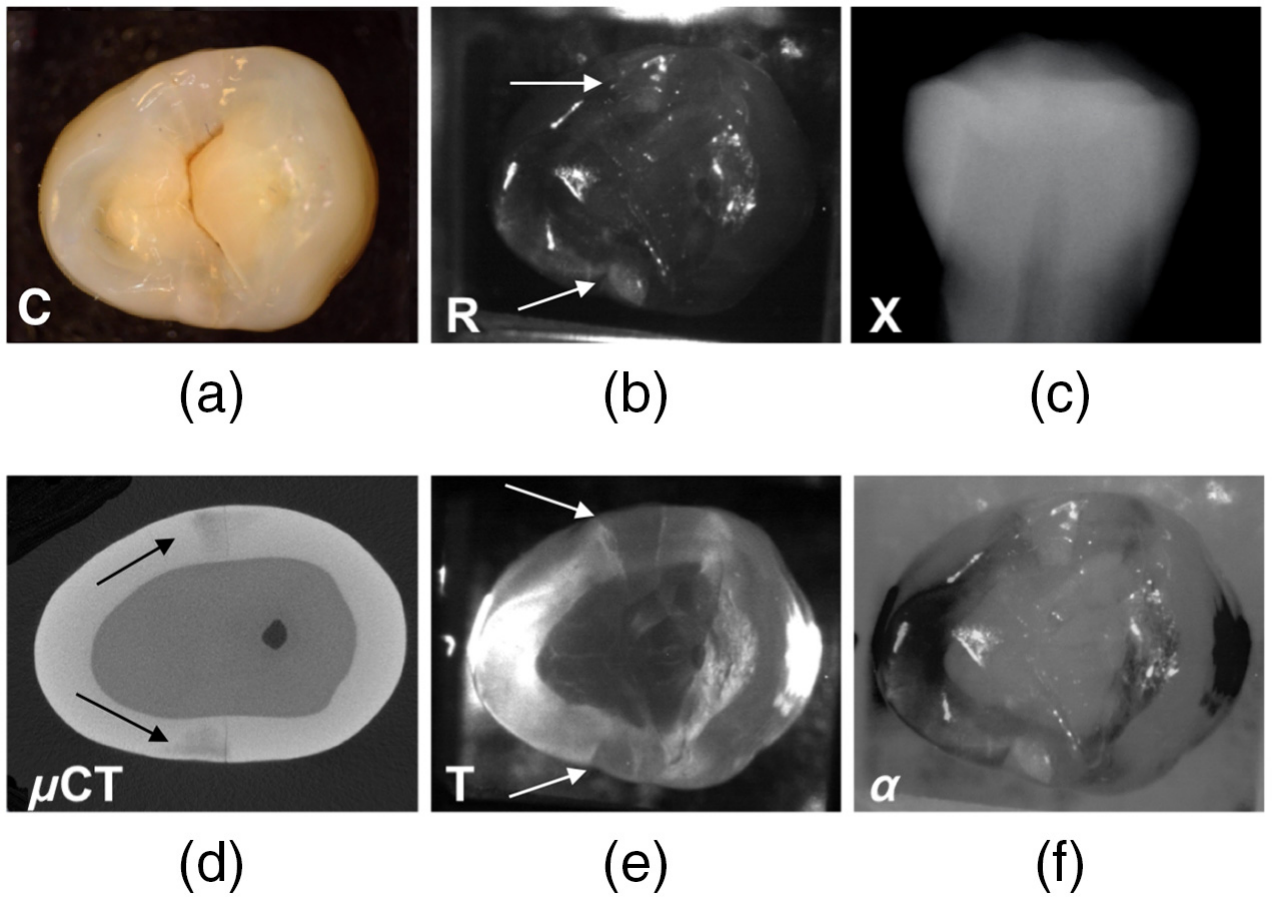
(a) Color; (b) SWIR reflectance at 1600 nm; (c) dental radiographic; (d) μ−CT; (e) SWIR transillumination at 1300 nm; and (f) combined R + T fusion (α=0.4) images of a tooth with two approximal lesions. Arrows point to lesions.

Several images of another tooth with approximal lesions at both proximal contact points are shown in Fig. 4. The visible image of the occlusal surface shows nearly no evidence of approximal lesions [Fig. 4(a)] and only one of the lesions is visible in the radiograph [Fig. 4(c)] and it appears to penetrate less than halfway through the enamel. The SWIR reflectance, transillumination, and μCT images agree quite well all showing that both lesions penetrate almost to the dentinal enamel junction (DEJ). The fusion image [Fig. 4(f)] shows the lesions with high contrast and the surrounding sound enamel areas appear more uniform in intensity.

**Fig. 5:**
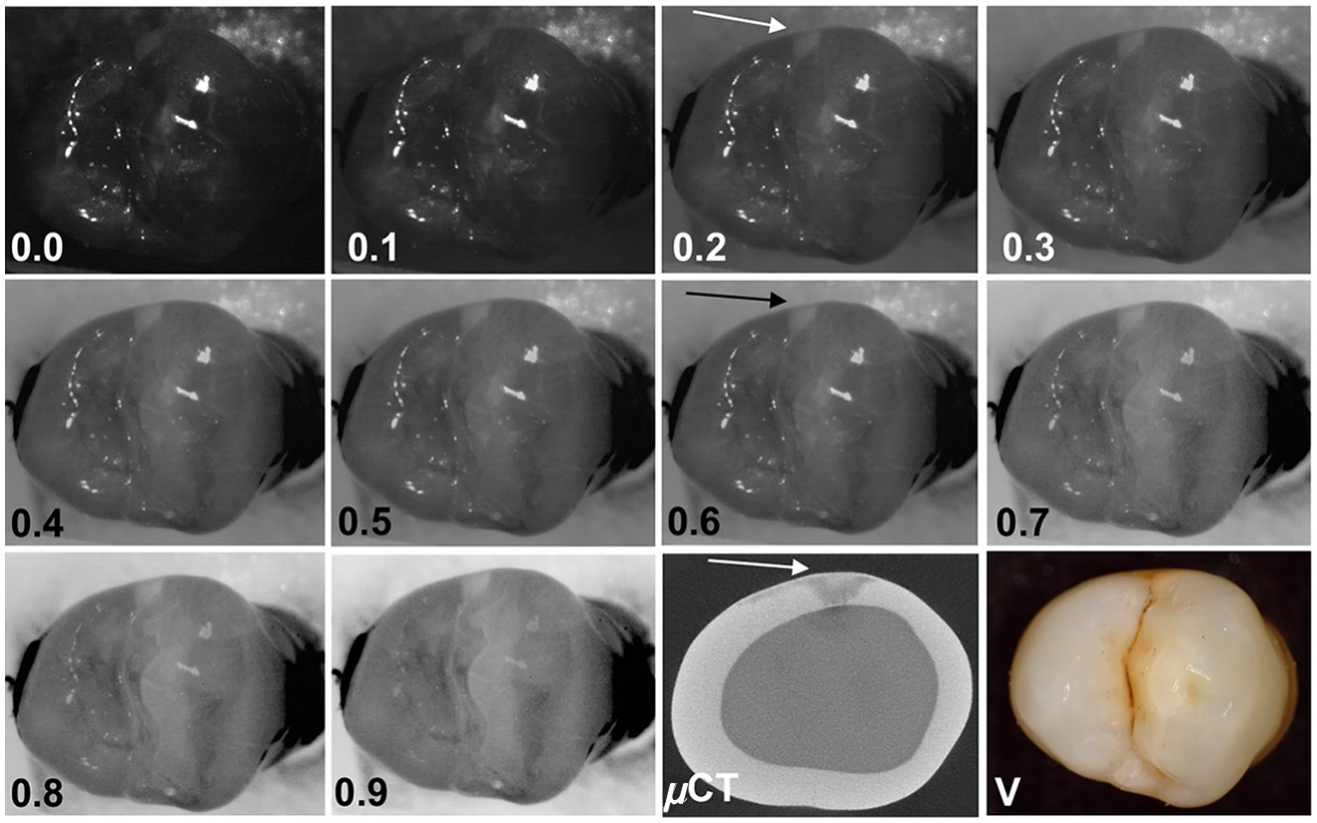
Combined transillumination and reflectance images with a varying from 0 to 0.9 along with visible (V) and an occlusal (μCT) μCT image of a tooth with occlusal decay. For this particular tooth the contrast increased with increasing a.

**Fig. 6:**
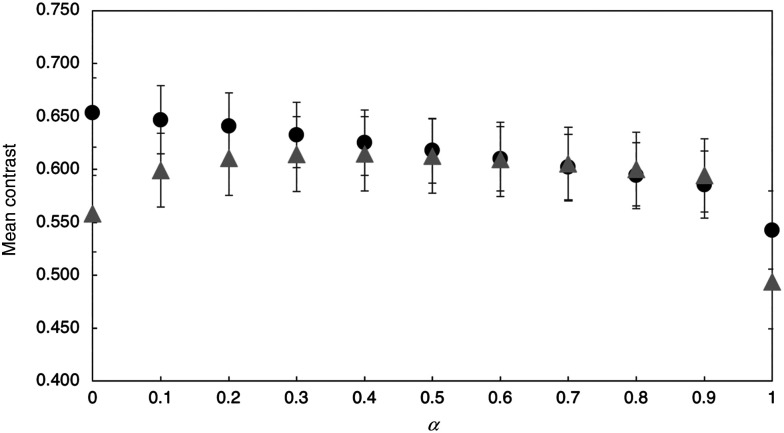
Plot of mean±SD of lesion contrast versus α for dual-probe fusion mode (α=0.4) images for occlusal lesions (circles, n=120) and approximal lesions (triangles, n=120).

Figure 5 shows the effect of varying alpha ($\alpha I_{\mathrm{Tinv}}+bI_{R}$) in the dual fusion images. Images with α values varying from 0.0 to 0.9 are shown of a tooth with an approximal lesion. For this particular tooth, the contrast of the lesion increased with increasing α. The contrast did not increase with increasing α for all of the teeth, some exhibited a decrease with increasing α. The mean lesion contrast from all samples is plotted as a function of the α coefficient in Fig. 6 for occlusal lesions (circles, n=120) and approximal lesions (triangles, n=120). The approximal lesion depth was measured from the proximal surface contact point to deepest lesion point in the pulpal direction while the lesion depth for occlusal lesions was measured from the occlusal surface to deepest lesion point in the pulpal direction.

**Fig. 7:**
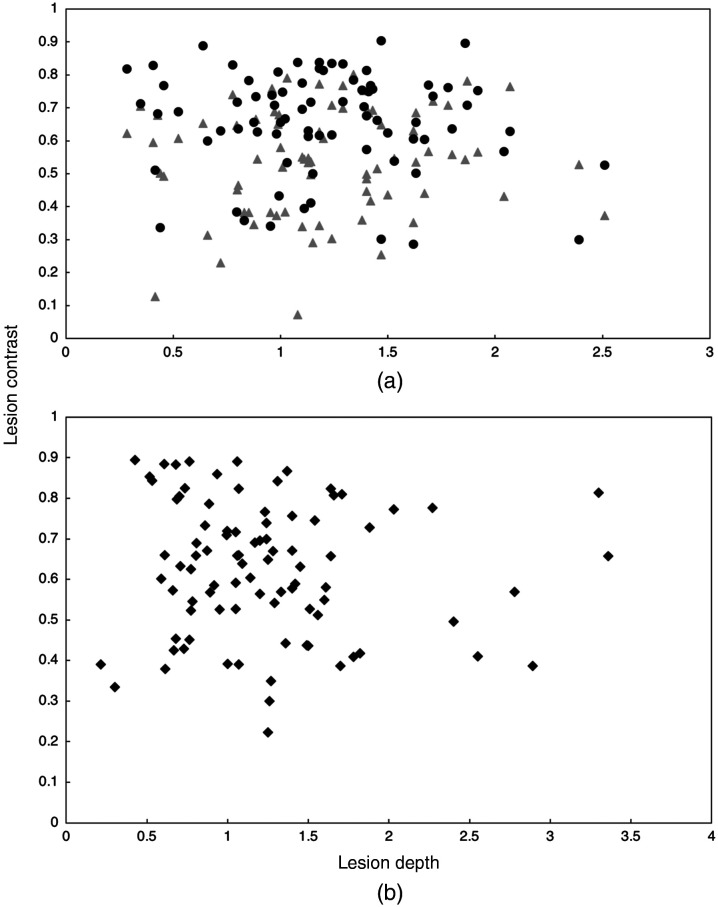
Occlusal lesion contrast versus lesion depth measured with μCT. (a) Reflectance (circles) and transillumination (triangles). (b) fusion mode images (α=0.4).

**Fig. 8:**
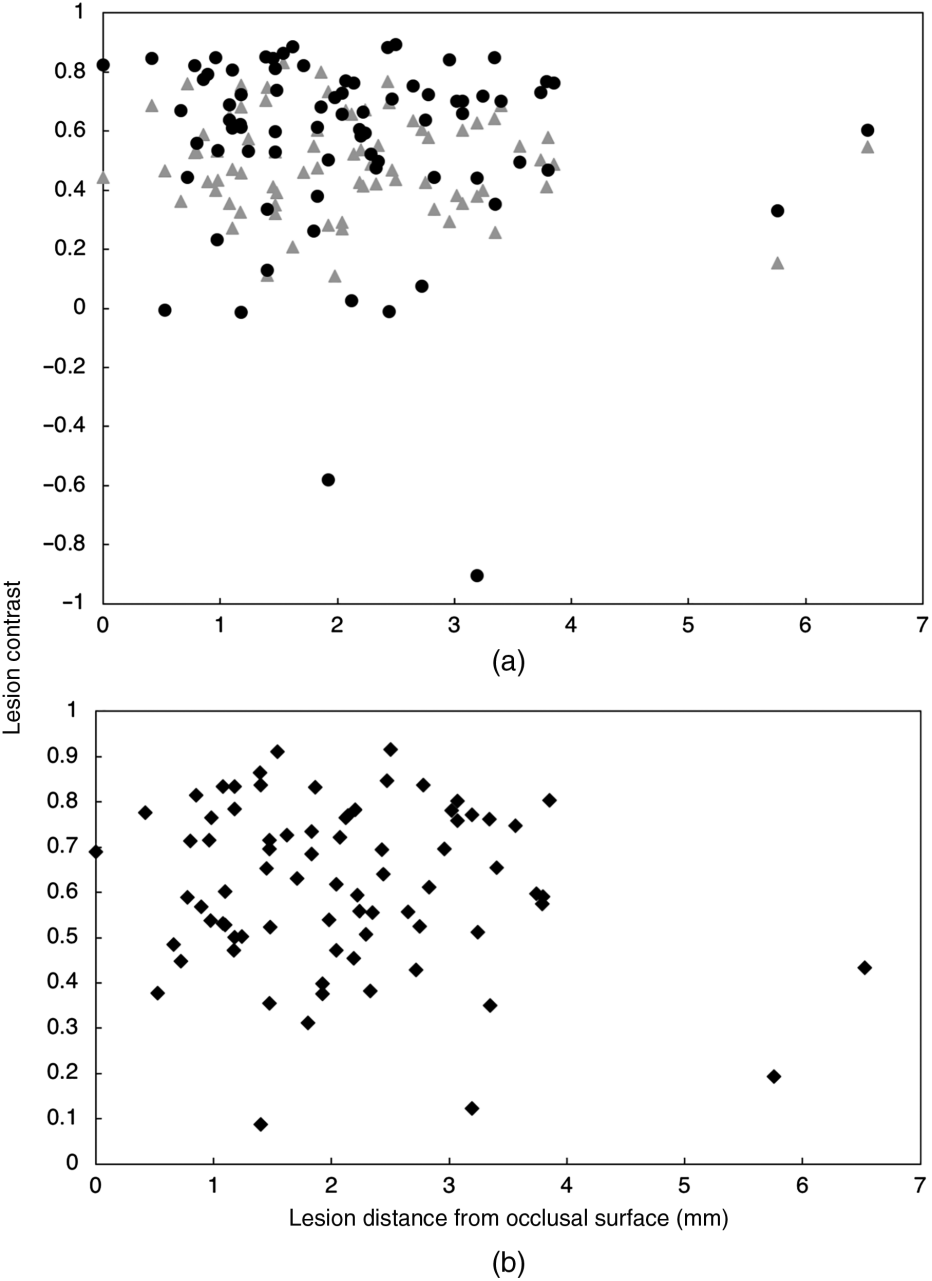
Approximal lesion contrast versus lesion distance from the occlusal surface measured with μCT. (a) Reflectance (circles) and transillumination (triangles). (b) fusion mode (α=0.4) images.

The mean lesion contrast is plotted versus lesion depth for occlusal lesions in Fig. 7 for reflectance, transillumination, and the fusion mode images. The reflectance and transillumination measurements were all from the occlusal surface. There was no significant dependence on the lesion contrast with lesion depth. For approximal lesions, the lesion contrast was determined versus the distance of the lesion from the occlusal surface. This comparison is shown in Fig. 8 for the reflectance and transillumination images along with the fusion images and there was also no significant dependence on the lesion contrast with distance from the occlusal surface.

The effect of α on the mean lesion contrast is tabulated in Table 1 for occlusal and approximal lesions. For occlusal lesions, the reflectance mode alone performs exceptionally as it shows a mean lesion contrast of 0.65 versus 0.57 from transillumination mode. Dual fusion mode negatively impacts the mean occlusal lesion contrast, and as α gets higher the mean lesion contrast decreases. For approximal lesions, α=0.4 shows a mean lesion contrast of 0.615, improving the contrast of 49.4% of the approximal lesions and showing contrast enhancement of 8.2% from the reflectance mode and 22.8% from the transillumination mode.

**Table 1:** Effect of α on the mean lesion contrast for occlusal and approximal lesions.

α	(α=0) R	0.1	0.2	0.3	0.4	0.5	0.6	0.7	0.8	0.9	(α=1) (T)
Occlusal lesion contrast	0.651	0.641	0.631	0.621	0.612	0.602	0.594	0.584	0.575	0.566	0.533
Approximal lesion contrast	0.558	0.599	0.611	0.614	0.615	0.613	0.610	0.605	0.6	0.595	0.494

The examiner agreement (percent agreement), accuracy, and FP rate are given in Table 2 for the three clinician assessments of the SWIR images and the radiographs. The mesial and distal proximal surfaces were combined for the table. The examiner agreement was highest for the radiographs. The examiner agreement was moderate for transillumination, reflectance, merged images, and side by side images for both occlusal and proximal surfaces. Reflectance yielded the highest accuracy and lowest FP rate while transillumination yielded the lowest accuracy and the highest FP rate among SWIR imaging. The use of dual mode yielded a slight improvement in accuracy, percent agreement, and FP rate over transillumination alone but was inferior to reflectance. The use of side-by-side reflectance and transillumination images did not appear to improve diagnostic performance or reduce the FP rate. Both the FP rate and the accuracy of radiographs were lower than the SWIR methods.

**Table 2:** Clinician scores for the occlusal and proximal surfaces of 106 teeth for percent agreement, accuracy, and FP rate for SWIR imaging methods and radiography.

Examiners	Method	Occlusal	Proximal
Percent agreement	Transillumination	0.75	0.76
	Reflectance	0.87	0.84
	Dual	0.80	0.79
	Side by side	0.82	0.80
	Radiography	0.88	0.91
Accuracy	Transillumination	0.54	0.45
	Reflectance	0.81	0.59
	Dual	0.71	0.53
	Side by side	0.70	0.55
	Radiography	0.36	0.49
FP rate	Transillumination	0.50	0.55
	Reflectance	0.31	0.36
	Dual	0.47	0.39
	Side by side	0.36	0.36
	Radiography	0.08	0.16

## 4 Discussion

In this study, a compact dual reflectance and transillumination SWIR imaging device was employed to acquire images of 120 extracted teeth with caries lesions. Near-simultaneous reflectance and occlusal transillumination images are collected from the tooth occlusal surface which facilitates registration and integration. The reflectance probe delivers 1600-nm light to the tooth occlusal surface while the occlusal transillumination probe delivers 1300-nm light cervically at the lingual and buccal sides of the tooth allowing it to propagate into the tooth diffuse in the cervical-coronal direction interacting with approximal and occlusal lesions and exiting the occlusal surface. The transillumination probe utilizes light at 1300 nm where the enamel is most transparent. Scattering in enamel continues to decrease with increasing wavelength beyond 1300 nm, however, water absorption increases markedly beyond 1400 nm greatly increasing attenuation.^34^ The dual SWIR probe can capture high definition *in-vitro* transillumination and reflectance SWIR images of extracted teeth. The probe was fabricated using a 3D printer and utilized two broadband fiber-optic SLD light sources. Previous studies have investigated the contrast of caries lesions using both reflectance and transmitted light. This was the first study demonstrating the performance of an imaging system that simultaneously acquires cross-polarized reflectance and occlusal transillumination images at multiple SWIR wavelengths using a single compact handpiece suitable for clinical use. In addition, it was the first study to compare the SWIR lesion contrast with μCT images as the gold standard for lesion depth.

The primary motivation for acquiring dual-mode images is to reduce the probability of FPs and remove the need to switch between transillumination and reflectance probes during screening. In SWIR occlusal transillumination, cracks and structural features in the tooth can block light propagation through the tooth producing features that resemble lesions, greatly increasing the potential for FPs. Specular reflection interferes with SWIR reflectance measurements and even when high extinction ratio polarizers are employed, there is still substantial specular reflection present in cross-polarization images that have the potential to generate additional FPs. Even though studies are lacking indicating that NIR imaging leads to an increased number of FPs, clinicians using this new technology have voiced concern regarding FPs. Moreover, the markedly higher sensitivity of NIR and SWIR imaging compared to radiography is likely to lead to an increase in FPs simply on the basis of the higher sensitivity. In our most recent clinical study involving SWIR imaging of teeth scheduled to be extracted with unknown caries status, the specificity of SWIR imaging was lower than for radiography, although the difference was not statistically significant.^9^ In that study images of the teeth were acquired using three separate imaging probes: SWIR reflectance (1600 nm), SWIR occlusal transillumination (1300 nm), and SWIR proximal transillumination (1300 nm), combining multiple images for diagnostic performance improved both the sensitivity and the specificity.

Another important advantage of imaging in the SWIR beyond 1150 nm is that the chromophores responsible for stains on teeth do not absorb light at longer wavelengths since there is not sufficient energy for electronic excitation.^1^^,^^26^ This is of particular importance in the stained pits and fissures of tooth occlusal surface. Ng et al.^15^ demonstrated that it is necessary to use SWIR wavelengths >1150  nm to avoid significant interference from stains when measuring lesion contrast in reflectance and transillumination modalities. Therefore, stains can be easily differentiated from actual demineralization in the SWIR range, which is not possible at visible wavelengths or at NIR/SWIR wavelengths of less than 1150 nm. Chung et al.^16^ showed that absorption due to stains contributed more to the lesion contrast than increased scattering due to demineralization at visible wavelengths and that is clearly demonstrated in Fig. 3. Since it is impractical and unnecessary to remove stains from the deep grooves and fissures on tooth occlusal surfaces, lack of interference from stains at longer SWIR wavelengths is a significant advantage.

Dental radiographs suffer from very low contrast, and it takes considerable experience and expertise to accurately read such images. The principal advantage of radiographs is the very high specificity, i.e., if a lesion is visible in a radiograph it is most likely a TP, therefore, overtreatment is less likely. In addition to the low sensitivity, radiographs typically underestimate the penetration depth of approximal lesions and dentists typically have to estimate that the lesions penetrate much deeper than they appear based on subjective factors involving caries risk status. Images acquired in this study suggest that SWIR images better represent the true depth of the approximal lesions and better match the depth indicated in μCT images than radiographs. SWIR images are also quantifiable and provide measurements that can be utilized for non-biased assessment of lesion depth and severity.

Both reflectance and occlusal transillumination imaging modes yield high contrast for approximal lesions. Since more light propagates through the transparent outer enamel than through the highly scattering dentin core, cracks, fractures, and other defects in the tooth can markedly influence light propagation in the tooth and can produce FPs. For example, two small fractures a few millimeters apart can produce a dark area on the outer ring of enamel that closely resembles an approximal lesion and this is the most common location for cracks in posterior teeth. The same cracks do not interfere with reflectance imaging since the light paths are directly above the lesion. Therefore, access to both reflectance and occlusal transillumination has the potential to increase the sensitivity and specificity of caries detection by optically sampling the entire external surface of existing lesions.

Reflectance imaging is more sensitive than occlusal transillumination for the detection of occlusal lesions. Even shallow demineralization confined to the outer half of the enamel can appear with high contrast in reflectance images. The challenge with SWIR imaging of occlusal lesions is differentiating the very shallow and superficial lesions from the lesions that penetrate through the enamel and into the underlying dentin. It is these deeper penetrating lesions that require removal and restoration. Remineralization therapy is a more appropriate treatment approach for shallow lesions. Previous studies have shown that the contrast increases significantly with increasing depth for SWIR reflectance imaging, however, the magnitude of the change is not sufficient to warrant the use of contrast thresholds to define lesion severity.^35^ Multiple studies including this one show that there is no dependence of the lesion contrast on depth for occlusal transillumination. A more reliable indicator of lesion depth and severity is the lesion size or width in the dimension perpendicular to the fissure or radial to a pit.^16^^,^^35^ Severe occlusal lesions typically arise in the plaque collection sites in the pits and fissures and grow from the base of the fissure to the underlying dentin. Upon reaching the dentin the lesion can spread more rapidly laterally along the dentinal-enamel junction. That lateral spread can be easily seen through the transparent sound enamel above the lesion, particularly at SWIR wavelengths and this approach of detecting these hidden occlusal lesions has been demonstrated *in vivo* using SWIR imaging and optical coherence tomography in two clinical studies.^36^^,^^37^ If the lesion becomes severe enough it can become visible to the naked eye as a dark shadow. In this study, there was no significant correlation of the lesion depth and the lesion contrast for either occlusal or approximal lesions for SWIR reflectance or transillumination. This contrasts with our earlier study utilizing SWIR reflectance^35^ where a significant correlation was observed between the lesion contrast and lesion depth for occlusal lesions. That prior study used optical coherence tomography to identify the occlusal lesions while this study used μCT, therefore, many of the lesions in the prior study were much shallower than the lesions identified in this study. This is one disadvantage of using μCT over optical coherence tomography, μCT is insensitive to the very shallow lesions that are commonly found on occlusal surfaces. The shallowest lesions imaged in this study were all greater than 0.5 mm in depth. In reflectance, the reflected intensity is expected to saturate above a given thickness/depth where it no longer increases with increasing depth.^38^ The thickness at which saturation occurs depends on the wavelength and the degree of demineralization. For greater lesion depths additional metrics are required for extrapolating potential lesion depth.

In addition to measurements of the lesion contrast, the SWIR images and radiographs were scored by three clinicians and μCT was used as a gold standard. Assessments of diagnostic performance should be carried out *in vivo* with a selection of teeth with a similar caries incidence expected for that population. That was certainly not the case in this study where all the teeth had lesions, however, the objective of this study was to show that the use of near-simultaneous SWIR reflectance and occlusal transillumination images has the potential for removing FPs and increasing diagnostic performance. Another concern with an *in vitro* study of this nature is that we have observed that *in vivo* transillumination images are typically better than *in vitro* images of lesions on extracted teeth due to the better internal hydration of vital teeth. The loss of internal water from extracted teeth markedly increases the scattering of sound dental tissues and reduces the lesion contrast.^16^ In addition, there were no overlapping or adjoining teeth, such teeth are expected to reduce the performance of radiographs and SWIR transillumination for imaging approximal lesions and would not be expected to influence the imaging of occlusal lesions or influence reflectance imaging. SWIR reflectance alone yielded the highest examiner agreement, highest accuracy, and lowest FP rate among the four image types. The SWIR reflectance alone had a higher accuracy than radiographs for approximal lesions (0.59 versus 0.49) and markedly higher accuracy for occlusal lesions (0.81 versus 0.36). The examiner agreement was highest for radiography and lowest for SWIR transillumination. This suggests that more training is required to read the SWIR images particularly the SWIR transillumination images. It was anticipated that either the dual mode or side by side SWIR images would yield the highest performance. However, the diagnostic performance for SWIR transillumination was quite low and that low performance most likely adversely influenced the dual mode and side by side images. It is likely that the performance of SWIR transillumination is most dramatically reduced in *in vitro* measurements due to the influence of the gingival tissues and the internal hydration of the tooth. The performance of this handpiece shown in Fig. 2(b) will be assessed *in vivo* over the next year and we will be able to confirm this hypothesis.

## 5 Conclusions

In summary, we have developed a clinical probe capable of acquiring near-simultaneous SWIR reflectance and occlusal transillumination images of approximal and occlusal lesions. The performance of the probe *in vitro* was assessed by imaging 120 extracted teeth with lesions on the occlusal and proximal surfaces. The next step will be to image *in vivo* teeth of unknown caries status scheduled for extraction for orthodontic reasons, to assess the diagnostic performance of this dual probe *in vivo*.

## Code, Data, and Materials Availability

There is no code essential for reproducibility or supplementary data available for this paper.
